# Lived experiences of cancer care for people living with HIV who are treated for anal cancer: a scoping review

**DOI:** 10.1136/bmjopen-2025-114180

**Published:** 2026-03-30

**Authors:** Christine Addington, Nigel Davies, Paul Howell, Susanne Cruickshank, Emma Hainsworth

**Affiliations:** 1 Health Services Research Unit, Royal Marsden NHS Foundation Trust, London, UK; 2 Brunel University London, Uxbridge, UK; 3 The David Adams Library, The Royal Marsden NHS Foundation Trust, London, UK

**Keywords:** HIV & AIDS, ONCOLOGY, Person-Centered Care, Quality of Life, Anus Neoplasms

## Abstract

**Abstract:**

**Objective:**

This scoping review aims to identify existing evidence on the lived experiences of people living with HIV and treated for anal cancer, and to identify what aspects of health and well-being are addressed in clinical guidance.

**Design:**

A preregistered protocol (Open Science Framework, 2025) guided the review. We followed the Arksey and O’Malley framework, incorporating Levac *et al*’s refinements around stakeholder consultation. Joanna Briggs Institute (JBI) guidance informed eligibility and data for charting, and reporting adhered to Preferred Reporting Items for Systematic Reviews and Meta-Analyses extension for Scoping Reviews guidelines.

**Data sources:**

Systematic searches were performed across multiple databases, including CINAHL, MEDLINE, PsycINFO and Embase, using EBSCOhost and Ovid, supplemented handsearching reference lists. Two search strategies were used: one for research studies and one for clinical guidelines.

**Eligibility criteria:**

Sources included people living with HIV treated for anal cancer, capturing lived experiences directly through qualitative studies or indirectly via quantitative patient-reported outcomes and/or health-related quality of life. Guidelines addressing HIV or anal cancer were also included.

**Data charting and summaries:**

Data were charted to capture patient experiences and outcomes on living with and beyond cancer, and how these are addressed in clinical management and guidance, including biomedical, psychosocial, sexual and functional aspects, and patient-reported outcomes.

**Results:**

Of 945 records, three studies and four guidelines met criteria. No study focused exclusively on people living with HIV; findings reflect broader anal cancer populations with HIV-positive subsets. Studies addressed aspects of health-related quality of life which we mapped into physical, psychosocial and sexual domains. Clinical guidance prioritised treatment dosage and survival, with limited attention to broader effects. Stakeholders highlighted that existing research and guidance miss important nuances of lived experience and care needs.

**Conclusions:**

No identified research solely explored the lived experiences of people living with HIV treated for anal cancer, leaving guidance non-specific and biomedical. The identified domains offer a starting point for future research; however, to inform patient-centred care, stakeholders emphasised the need to understand how living with HIV and anal cancer shapes health needs

**STRENGTHS AND LIMITATIONS OF THIS STUDY**

- Followed a pre-established protocol with frameworks and search strategy developed with a librarian.
- Collaborated with experts by experience.
- Inclusion of both research studies and clinical guidance.
- No formal quality appraisal conducted.
- Insights into lived experiences may be a feature of some grey literature which has not been captured within this search strategy.

## Background

 Current UK healthcare services face challenges in providing the person-centred care essential for those living with HIV and anal cancer. Advances in HIV treatment have provided the chance for people with HIV to live longer, but this may also increase the risk of developing multiple comorbidities including cancer.^1^ Although anal cancer is rare, affecting around 1600 people annually in the UK,^2^ it disproportionately impacts individuals living with HIV, particularly men who have sex with men.^3^ The unique health challenges faced by people living with HIV who are undergoing treatment for anal cancer remain poorly understood—particularly where multiple, intersecting issues compound one another. Evidence from other areas of healthcare highlights several key concerns: the physical toxicities associated with cancer treatment,^4^ the complexity of navigating care across multiple medical specialties,^5^ and the emotional burden of managing HIV-related stigma while accessing unfamiliar cancer services.^6^ Taken together, these factors may place people living with HIV at increased risk of poorer health outcomes during and after anal cancer treatment.

While the prognosis for anal cancer is generally favourable, with around 70% of people aged 15–44 surviving beyond 10 years and 55%–65% of people over 50 surviving 5 years,^2^ treatment with chemoradiotherapy can result in both short-term and long-term side effects. These side effects may affect the skin, bowel, bladder and sexual function and can lead to considerable psychological and social impacts on patients’ lives.^4^ Comparative studies in broader oncology suggest that people living with HIV report more frequent and severe symptoms during cancer treatment,^7^ implying that the consequences of cancer treatment on physical, psychological and social well-being may be greater in this population.

Understanding the consequences of treatment while both living with and beyond cancer is essential for healthcare providers and policymakers to develop patient-centred driven guidelines.^8 9^ Health-related quality of life (HRQoL) and other patient-reported outcomes (PROs) can help to capture the broader physical, psychosocial and sexual consequences of treatment.^10^ However, while recommendations for the adoption of HRQoL measures are acknowledged in clinical oncology trials to support labelling claims, reduce treatment burden and support treatment decision-making,^11^ their use in anal cancer remains limited.^12^ Clinical trials typically focus on biomedical outcomes such as survival, tumour response and treatment toxicity.^9^ These gaps in HRQoL measurement are particularly concerning for people living with HIV, whose lived experiences, values and support needs while undergoing cancer treatment are often overlooked or insufficiently understood.^6^ To create services that fully support individuals with their healthcare needs, it is crucial to prioritise the expertise of people living with HIV who have been treated for anal cancer.

The aim of this scoping review was to identify the evidence on the lived experiences of cancer care for people living with HIV who are treated for anal cancer: to gain an overview of the research in this field and identify gaps for future work. The insights gained from this review will help guide and shape future exploratory and co-production efforts focused on improving service pathways. This review is part of a larger programme of work aimed at co-producing an intervention to support individuals living with HIV who are being treated for anal cancer.

## Methods

### Protocol and registration

This scoping review follows a pre-established protocol registered with the Open Science Framework (OSF) on 9 January 2025 (https://doi.org/10.17605/OSF.IO/F7YJZ). The methodology followed the six-stage framework by Arksey and O’Malley,^13^ incorporating Levac *et al*’s refinements, including stakeholder consultation.^14^ JBI guidance^15^ informed eligibility criteria and the data charting process, and reporting adhered to Preferred Reporting Items for Systematic Reviews and Meta-Analyses extension for Scoping Reviews (PRISMA-ScR) guidelines^16^ (see online supplemental PRISMA-ScR checklist).

### Research question identification

Previous unpublished service improvement work by author EH informed the development of the scoping review topic and through iterative discussions, the scope and review question were refined. Key concepts were explored through mind maps, focusing on two areas: (1) lived experience of receiving treatment for anal cancer and (2) anal cancer policy and guidelines, both within the context of living with HIV. These mind maps allowed for the exploration of key terms that would be later used for the database search.

For this scoping review, we operationalised ‘lived experience’ as individuals’ direct experience of individuals’ perceptions and everyday healthcare realities of being treated for anal cancer while living with HIV,^17^ encompassing experiences related to diagnosis, treatment, life beyond treatment, and physical, functional, and psychosocial aspects. Although lived experience research typically relies on qualitative, inductive approaches,^18^ no exclusively qualitative studies were identified in this population. We therefore included quantitative studies reporting PROs, symptom burden, functional status or quality of life measures as indirect evidence of lived experience.^19^ Questionnaire-based measures were considered appropriate because they capture patients’ perceptions and daily healthcare realities, providing structured insight into experiences that qualitative research would typically explore.^19^ Inclusion of these measures also allowed comparison with our consultation group’s treatment priorities, highlighting potential discrepancies between contemporary research focus and patients’ lived experiences.

Clinical guidelines were included to help determine whether current clinical practice recommendations reflect elements of patients’ experiences and needs beyond survival or disease control. This included consideration of treatment-related functional impairment, sexual health, psychosocial well-being, long-term survivorship and needs specific to people living with HIV.

### Search strategy

We collaborated with a specialist healthcare librarian (PH) to develop and refine our search strategy. To capture both original research and clinical documents and policies, we created two distinct search strategies. The first strategy focused on primary research papers, including both experimental and exploratory studies. The second strategy targeted clinical guidelines and policy documents, ensuring the inclusion of authoritative sources that inform clinical management.

The overall structure of both search strategies followed the Population, Concept and Context (PCC) framework^16^:

- **Population:**People living with HIV and diagnosed with anal cancer.
- **Concept**: Quality of life, person-centred care, psychological impact, PROs, survivorship and satisfaction with care.
- **Context**: Individuals who have received or are undergoing treatment for anal cancer, including radiotherapy, chemotherapy and/or surgery.

The search strategy, with relevant keywords and index terms, was adapted for each database and applied to CINAHL, Psychology and Behavioral Sciences Collection (via EBSCOhost); MEDLINE and PsycINFO (via Ovid); and Embase, Emcare, HMIC, Social Policy and Practice, Royal Marsden Journals (via Ovid). The initial search was conducted from November 2024 to January 2025, covering each database from inception to present and was rerun in November 2025 to identify any new publications. The full search terms and strategies are listed in supplemental document 2. Boolean operators were used: AND between population, concept and context terms, and OR between synonyms.

Due to the anticipated small number of studies, as discussed with the librarian, separate searches were conducted for each concept within the PCC framework (eg, quality of life, lived experience, person-centred care). This approach aimed to increase the search sensitivity, broaden coverage and minimise the risk of missing relevant studies. An example of this is provided for the concept of quality of life in table 1.

**Table 1:** Example of search terms and synonyms applied for the concept ‘Quality of Life’ in database searches

Population	Context	Concept
	**And**	**And**
Human Immunodeficiency Virus	Anal cancer	Quality of Life
**OR**	**OR**	**OR**
HIV-positive	Anal carcinoma	Health-related quality of life (HRQoL)
People living with HIV	Anal squamous cell carcinoma	Health-related life quality
HIV	Adenocarcinoma of the anus	Life quality
	Anal malignancy	Satisfaction
	Anal tumours	Psychosocial well-being
	Anal neoplasms	Patient-reported outcomes

To identify clinical guidelines, the term ‘guidelines’ (and its synonyms) was included in the search string, along with searches via Google Scholar and relevant charity and organisational websites.

For both the study and clinical guideline search, the reference lists of eligible articles were handsearched for further relevant literature.

### Eligibility criteria

The requirements of the scoping review were created from the PCC framework (see table 2). No country-related limitations were applied. However, as our future co-development work would be taking place within the UK, the clinical guidelines were limited to the UK. While no time frame was applied to the search for primary research due to the small number of anticipated outputs, a 5-year limit was applied to policy documents and clinical guidelines to capture the most current and relevant guidance.

**Table 2:** PCC-based eligibility criteria for the scoping review (people living with HIV, treated for anal cancer, lived experience)

Inclusion	Exclusion
Qualitative, quantitative or mixed methods providing insights into the experiences of patients treated for anal cancer while living with HIV	Sole focus on medical or clinical outcomes without exploring patient experiences
Studies including the lived experiences of people treated for anal cancer—key aspects (eg, lived experience, quality of life, satisfaction, healthcare needs)	Preclinical studies, laboratory research or animal studies
No time frame for research and last 5 years for policy and clinical guidelines	Exclusive focus on HPV screening, anal intraepithelial neoplasia, or general epidemiological findings
	No assessable English translation
	HIV alone or anal cancer alone (unless they explore combined experiences or provide relevant insights)

HPV, human papillomavirus; PCC, Population, Concept and Context.

As is standard in scoping review design, post hoc, eligibility criteria were iteratively refined and applied based on familiarity with the topic gained through reading and engaging with the literature.^14^ Studies reporting only biomedical outcomes (eg, tumour response, survival or toxicity rates) were excluded, as these do not provide patient-reported, functional or experiential data relevant to this review.

#### Clinical guideline eligibility criteria

- Included if the guideline provided recommendations relevant to the treatment, care, or management of anal cancer, regardless of whether HIV was explicitly addressed.
- Included if the guideline focus was on HIV but contained guidance related to general cancer care (ie, across tumour types), including screening, diagnosis, treatment, survivorship or supportive care.
- Excluded if the guideline focused on HIV without any content related to cancer.

### Study selection

All citations from each database search were imported into RefWorks (ProQuest, 2021), where duplicates were removed. The first author then handscreened the titles and abstracts for relevance. Our search combined terms for anal cancer and HIV, but titles or abstracts did not need to mention HIV. This allowed us to capture all anal cancer studies with terms related to HRQoL, lived experience or person-centred care, and then review full texts for any HIV subgroups. After that, full-text screening was initially conducted by the author CA, and 10% of the papers were evaluated by EH. The final set of papers was discussed by the CA and EH and then presented to the wider research team, which included experts and individuals with lived experience, to identify any potentially missed articles.

### Data charting

Two separate data charts were made in Excel (Microsoft, 365) for the two search strategies. Charted information included author, year, country, type of study, outcome measures, key concepts and main findings.

### Collating and summarising

During the data charting phase, findings were organised under key headings, with each category illustrating the issues, experiences and/or policy-related factors affecting people living with HIV undergoing treatment for anal cancer. The descriptive summaries outlined the aims, methods and key findings of the included studies and policies in relation to the scoping review questions. By comparing the content of clinical guidelines and research studies with reported patient experiences and outcomes, the review aimed to identify where practice aligned with or diverged from actual care. This comparative approach provided insight into the relationship between clinical guidance, treatment delivery and the needs of this patient population.

### Patient and public involvement

To ensure the scoping review addressed meaningful issues with sensitivity and relevance, people and public contributors with lived experience of HIV and cancer informed the review focus and interpretation of findings. A 1-hour online workshop was held with CA, EH, ND and three stakeholders (including individuals with lived experience) who had consented to support the review. No formal training was provided; contributors had relevant experience through prior service evaluation work with EH. During the workshop, creative reflection and critical discussion were used to consider how the preliminary research findings and current clinical guidelines aligned with stakeholders’ lived experiences and to identify gaps that could enhance support for anal cancer treatment (see online supplemental file for workshop resources). Contributors were reimbursed in line with best practice guidance.^20^ An anonymous post-workshop feedback survey was used to evaluate Patient and Public Involvment and Engagement (PPIE) activites and identify areas for improvement.^21^

## Results

### Searches

**Figure 1:**
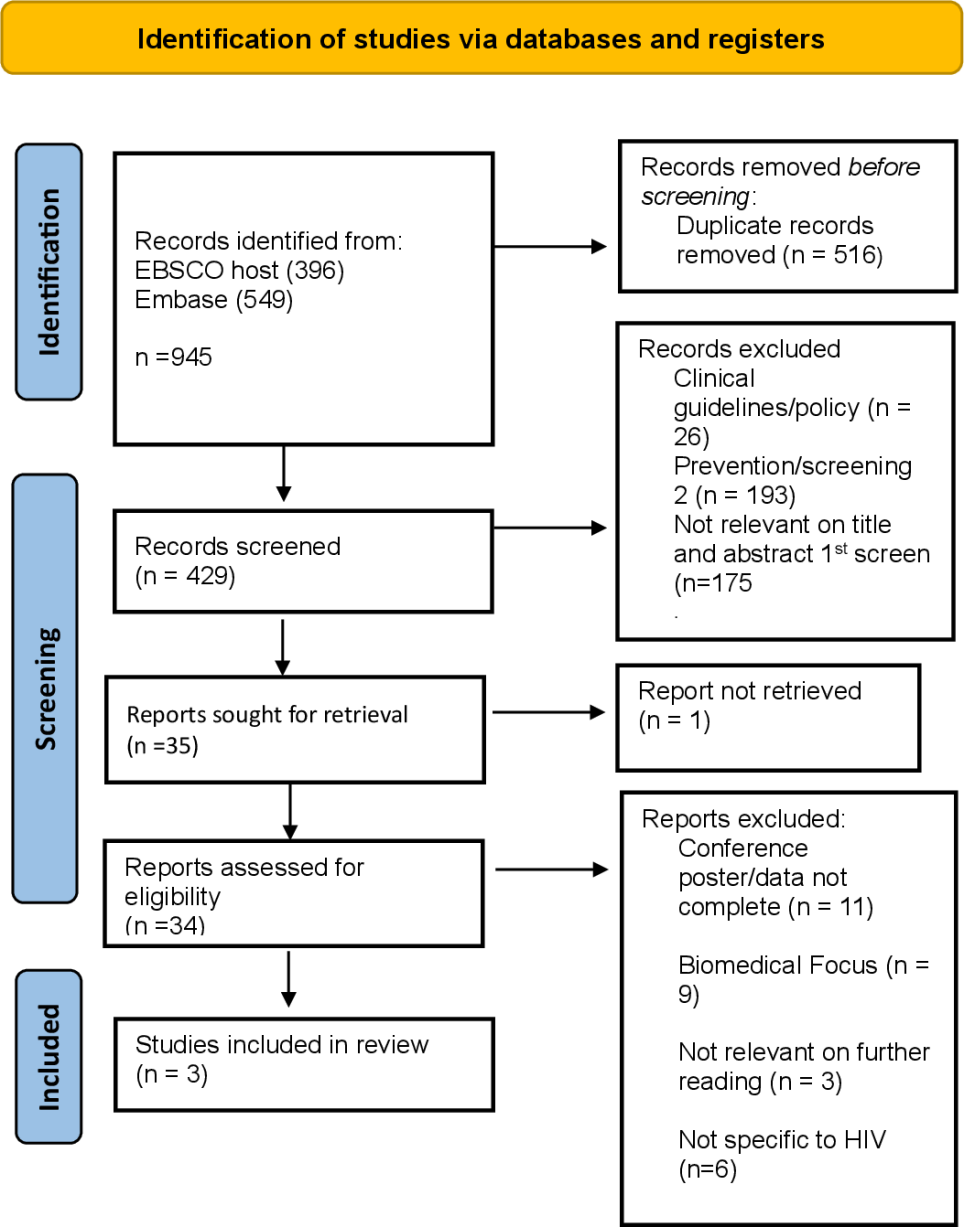
PRISMA flow chart for strategy 1. Finding research papers for PCC. PCC, Population, Concept and Context; PRISMA, Preferred Reporting Items for Systematic Reviews and Meta-Analyses.

The study search retrieved 945 records, of which 516 duplicates were removed. Title and abstract screening of the remaining 429 records identified 34 for full-text retrieval. Following full-text screening, three articles were included in the final review (see figure 1). For relevant clinical guidelines and policy, database and hand searching retrieved 26 records, of which four met the scoping review requirements.

### Description of studies (design, population and aims)

Table 3 summarises the study design, population, aims, outcomes and key findings of the three included studies. All included studies were observational and survey-based, but used fundamentally different approaches: one prospective study,^22^ one cross-sectional survey^23^ and one secondary analysis^24^ of a national Lesbian, Gay, Bisexual and Transgender (LGBTQ+) cancer survey.^25^

**Table 3:** Summary of study characteristics; aims, participant description, outcomes and tools and main findings

Author	Year	Country	Research design	Aim	Demographics of popn	Outcome of interest	Outcome tools	Results
Mauro *et al*^22^	2021	Brazil	Prospective survey study Single centre	To assess quality of life and sexual function among men who have sex with men following treatment for AC	Total sample: n=19 MSM: n=19 Living with AC: n=19 Living with AC and HIV: n=15 Received chemoradiation: Yes Median age: 59 years	Quality of life Sexual function	EORTC QLQ C30^26^ IIEF^27^ HAT-QOL^28^ Sexual inventory^22^	QLQ-C30 scores showed a declining trend at end of treatment and 3 months, with improvement by 6–12 mo; but only emotional functioning declined significantly (p=0.048).
Challabout *et al*^23^	2023	USA	Cross- sectional survey Single centre	To compare patients’ expectations of radiotherapy for AC with their actual treatment experiences, with a particular focus on physical symptoms and stigma	Total sample: n=46* Male: n=13 Female n=20 Living with AC: n=46 Living with AC and HIV n=4 Received chemoradiation Median age: 66 years	Baseline knowledge and expectations about treatment vs actual experience Quality of Life (specific toxicities) Experience of stigma	ARAC^23^ EORTC QLQ-ANL27 (NR) EORTC QLQ-CR29 (NR) DISC-12^29^	73% reported little to no pretreatment knowledge of chemoradiation; 70% reported short-term effects were worse than expected, 39% reported worse-than-expected long-term effects; a subset concealed their diagnosis and withdrew from close relationships
Franco-Rocha *et al*^24^	2024	USA	Secondary analysis of national LGBTQ+ cancer survey data	To understand psychosocial health of sexual and gender minority individuals with GI cancers and identify differences in health outcomes among different cancer types	Total GI group n=295 total sample AC group n=133 Male n=122 Female n=9 Living with AC n=133 Living with AC and HIV: not recorded Treatment details: not recorded Median age: 60 years	Psychosocial health (mental health and social health) Impact of cancer on identity and intimate relationships	Questions within OUT: The National Cancer Survey^24^ (including free text)	46% received LGBTQ+-tailored mental health resources, though 81.9% saw them as valuable; more days of poor mental health were linked to current cancer, disability and fewer close friends; cancer and treatment negatively impacted intimacy, with free text highlighting effects on physical and mental health

We use ‘LGBTQ+’ to refer inclusively to the broader LGBTQ+ community, which includes people who identify as queer, intersex, asexual, and others with diverse gender identities and sexual orientations

* (Response rate was 33/46). NR—means that the year or version of the outcome measure was not recorded by the authors.

AC, anal cancer; ARAC, assessment of knowledge and comparison questions; DISC-12, Discrimination and Stigma Scale (12-item version); EORTC QLQ-ANL27, EORTC QLQ Anal Cancer–specific Quality of Life Module (27 items); EORTC QLQ-C30, European Organisation for Research and Treatment of Cancer Quality of Life Questionnaire (Core 30-item version); EORTC QLQ-CR29, EORTC QLQ Colorectal Cancer–specific Quality of Life Module (29 items); GI, gastrointestinal; HAT-QoL, HIV/AIDS-Targeted Quality of Life Instrument; IIEF, International Index of Erectile Function; LGBTQ+, Lesbian, Gay, Bisexual, and Transgender; MSM, Men who have sex with men; OUT Survey, ‘OUT, The National Cancer Survey’ (focused on LGBTQ+ individuals with cancer.

Mauro *et al*^22^ focused on sexual function and general cancer-related quality of life among men who have sex with men, treated for anal cancer. Chaballout *et al*^23^ explored treatment expectations, physical symptoms and stigma in a mixed sample of men and women. Franco-Rocha *et al*^24^ investigated psychosocial health across gastrointestinal cancers, including anal cancer, among sexual and gender minority individuals. None focused exclusively on people living with HIV treated for anal cancer, but all included HIV-positive participants: Mauro *et al*^22^ 79% (n=15), Chaballout *et al*^23^ 12% (n=4) and Franco-Rocha *et al*^24^ an unspecified number (HIV status was not systematically captured, but qualitative responses indicated some participants were HIV-positive).

### Outcomes

The three studies employed different approaches to assess treatment impact on HRQoL (table 4). Mauro *et al*^22^ and Chaballout *et al*^23^ used validated multidimensional questionnaires, while Franco-Rocha *et al*^24^ analysed data from a national survey. Across the studies, outcomes included cancer-related quality of life, anal cancer-specific quality of life, sexual function, stigma, treatment expectations, healthcare access, and psychosocial and relationship factors (outcome measures and the domains captured can be found in table 2).

**Table 4:** Domains reported by outcome measures across three studies: physical, sexual, psychosocial and HIV-specific

		Physical toxicities					Physical function					Sexual function			Psychosocial health						
		Bowel issues	Anal pain/discomfort	Skin toxicity	Urinary issues	Abdo pain/bloat	Stoma	General cancer toxicity	General cancer impact	Anal cancer-specific impact	HIV-specific impact	Male	Female	Sexual activity	Body image	Stigma	HIV-specific stigma	General cancer social	HIV- specific social	General cancer mental health	HIV- specific mental health
Challabout *et al*^23^																					
	EORTC ANL27	✔	✔	✔	✔	X	✔	X	X	✔	X	✔	✔	✔	X	X	X	X	X	X	X
	EORTC CR29	✔	✔	X	✔	✔	✔	✔	X	X	X	✔	✔	X	✔	X	X	X	X	✔	X
	DISC-12	X	X	X	X	X	X	X	X	X	X	X	X	X	X	✔	X	X	X	X	X
	ARAC	✔	✔	✔	✔	✔	X	✔	X	✔	X	✔	✔	✔	✔	✔	X	✔	X	✔	X
Mauro *et al*^22^																					
	EORTC QLQ-C30	✔	X	X	X	X	X	✔	✔	X	X	X	X	X	X	X	X	✔	X	✔	X
	HAT-QOL	X	X	X	X	X	X	X	X	X	✔	✔	✔	X	X	✔	✔	X	✔	X	✔
	IIEF	X	X	X	X	X	X	X	X	X	X	✔	X	X	X	X	X	X	X	X	X
	Sexual inventory	X	X	X	X	X	X	X	X	X	X	X	X	✔	X	X	X	X	X	X	X
Franco-Rocha *et al*^24^																					
	OUT Survey	X	X	X	X	X	X	X	X	X	X	X	X	X	X	X	X	✔	X	✔	X
	OUT Survey (FT)	X	X	X	X	X	X	X	X	X	*	*	*	*	*	*	*	*	X	*	X

A tick (✔) indicates item included in the outcome measure, a cross (X) indicates item not included in the outcome measure and an asterisk (*) indicates mentioned only within a qualitative quote, that is, it was not listed as part of a direct question. Note: As Challabout *et al*^23^ did not provide a date for their EORTC questionnaires, we used the most recent version available.^47 48^

ARAC, assessment of knowledge and comparison questions; DISC-12, Discrimination and Stigma Scale (12-item version); EORTC QLQ-ANL27, EORTC QLQ Anal Cancer–specific Quality of Life Module (27 items); EORTC QLQ-C30, European Organisation for Research and Treatment of Cancer Quality of Life Questionnaire (Core 30-item version); EORTC QLQ-CR29, EORTC QLQ Cancer–specific Quality of Life Module (29 items); HAT-QoL, HIV/AIDS-Targeted Quality of Life Instrument; IIEF, International Index of Erectile Function; OUT Survey, OUT, The National Cancer Survey; OUT Survey (FT), free text questions.

Notably, only Mauro *et al* included an HIV/AIDS-Targeted Quality of Life measure (HAT-QoL); otherwise, outcomes across all three studies were analysed in aggregate, pooling participants with anal cancer only and those living with both anal cancer and HIV. However, qualitative responses from Franco did allow identification of some HIV-specific experiences.

For all participants, Mauro *et al*^22^ recorded a baseline sexual inventory, the European Organisation for Research and Treatment of Cancer Quality of Life Questionnaire (Core 30-item version) (EORTC QLQ-C30) (general cancer-related quality of life including functional status and symptom burden),^26^ and the International Index of Erectile Function (IIEF) (sexual function including erectile function, orgasmic function, sexual desire, intercourse satisfaction and overall satisfaction).^27^ In addition, participants living with HIV also completed the HAT-QoL.^28^ All questionnaires were compared from baseline to post-treatment.^22^ In contrast, Chaballout *et al* used a merged questionnaire (EORTC QLQ-ANL27 (NR), QLQ-CR29 (NR) and Discrimination and Stigma Scale 12-item version)^28 29^, with data collected at a single post-chemoradiotherapy follow-up time point and analysed by comparing response frequencies for each question.

Franco-Rocha *et al* selected quantitative data on demographics, clinical characteristics, psychosocial health and mental health resource access to identify predictors of poor mental health days, alongside qualitative responses exploring identity and relationships. Quantitative and qualitative findings were analysed separately (multiple regression and thematic analysis, respectively), then integrated through narrative ‘weaving’. This generated three overarching themes: (1) factors that supported or undermined participants’ psychological well-being; (2) ways participants adjusted their social relationships with friends, partners and family and (3) other influences on psychosocial well-being during and after cancer.^24^

### Descriptive summaries

**Figure 2:**
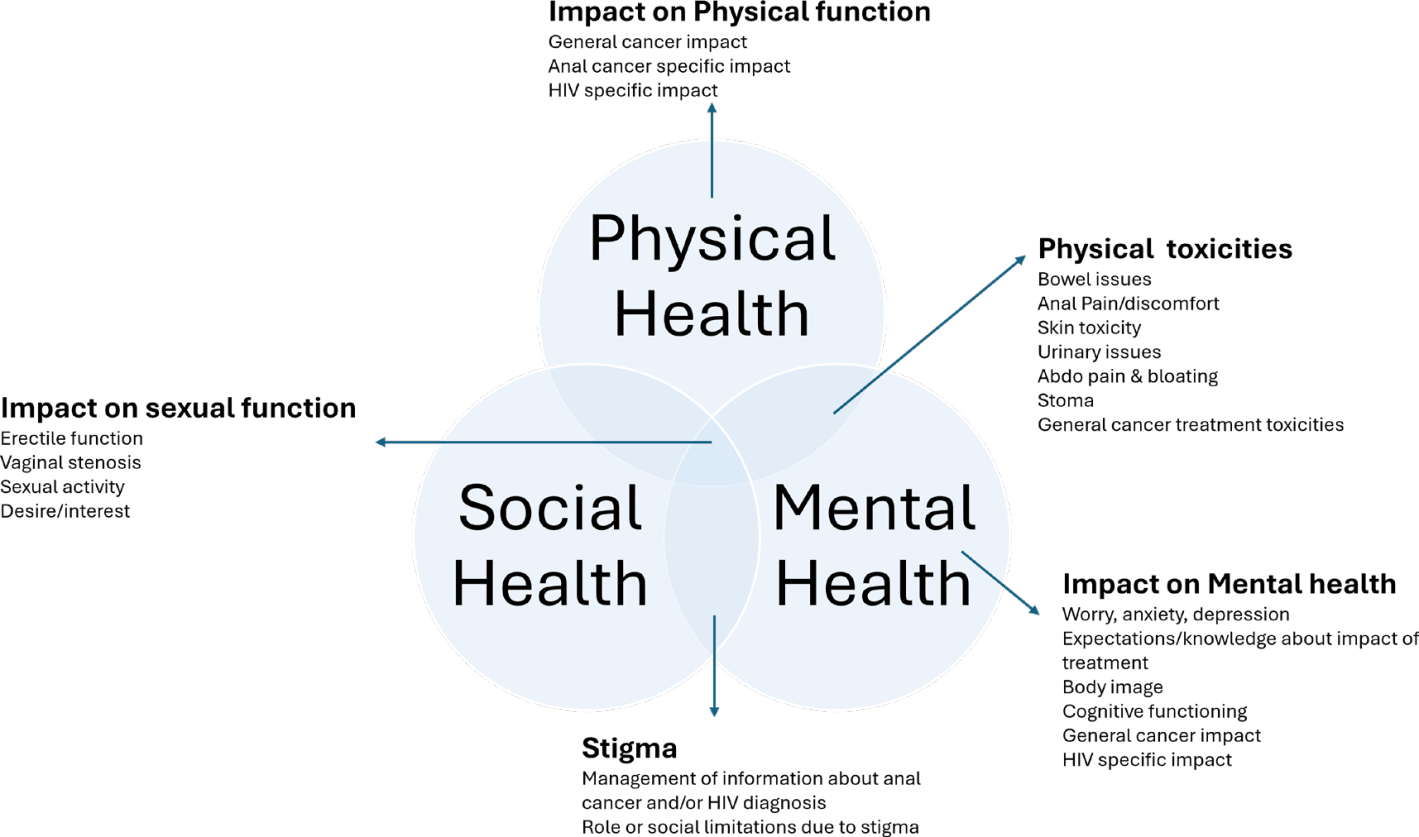
Illustration of three core domains of health-related quality of life—physical, mental and social—identified among the review papers and mapped the areas investigated across the included studies within each domain. Adapted from van Leeuwen *et al.*^49^

Key elements of lived experience, reflected in quality-of-life measures, were grouped into three broad, overlapping themes. See figure 2 for an illustrative overview.

- Physical health—for example, toxicities from treatment/disease and impact on physical function.
- Psychosocial health—comprising mental and social health (including stigma).
- Sexual health—impact on interest, activity and function.

#### Physical health: toxicities from treatment/disease and impact on physical function

Symptoms such as pain, nausea and fatigue were reported across the three studies. Mauro *et al* observed no significant differences in physical toxicities or physical functioning from baseline to 12 months post-treatment.^22^ In contrast, Chaballout *et al* found that 70% of participants reported worse-than-expected short-term treatment effects, particularly regarding bowel habits, with 82% identifying bowel issues as a major concern.^23^ Long-term, 73% reported more bowel-related side effects than anticipated.^23^ Franco-Rocha *et al* did not report quantitative physical health data, however, qualitative comments noted concerns about the impact of cancer treatment on immune function in people living with HIV:

Cancer surgery was a nightmare. Being HIV+ I am more keenly aware of the very negative impact that radiation has had on my immune system. ^24^

#### Psychosocial health: comprising mental and social health, including stigma

Mauro *et al* found no statistically significant changes in QLQ-C30 domains from the end of treatment through the 12-month follow-up period, except for the emotional functioning domain (p=0.048).^22^ While emotional well-being scores appeared to improve, the authors did not report post hoc analyses, making it unclear at which specific time points differences emerged. At 6-month and 12-month follow-up, Mauro observed a strong positive association between QLQ-C30 and HAT-QoL scores; however, p values, sample size and correlation coefficients were not reported. The authors suggest this indicates similarity in quality of life between participants living with HIV and those without, but no separate analyses were provided to support this claim. This correlation is expected, as the same participants completed both questionnaires, with only four participants not living with HIV (who did not complete the HAT-QoL). Therefore, the correlation indicates only that the two questionnaires capture similar response patterns.

Chaballout *et al* reported that between 12%–30% of participants had concealed their diagnosis or treatment side effects from friends, family or colleagues, and 15% indicated that stigma had led them to avoid close relationships.^23^ Franco-Rocha *et al* reported that only 46% of respondents with anal cancer had received LGBTQ+-specific mental health resources, despite 82% expressing a need for them.^24^ Those with current cancer diagnoses, disabilities or fewer close relationships were associated with a higher number of poor mental health days.^24^ Qualitative data from this study illustrated experiences of shame and isolation, with one participant linking anal cancer directly to feelings of stigma and secrecy, stating:

The fact that it’s ANAL cancer shames me. So, I stay isolated about it. Not worrying family.^24^

#### Sexual health: impact on interest, activity and function

Sexual health was most comprehensively addressed in Mauro *et al* ; however, despite reported variation among measures of sexual well-being and increasing prevalence of sexual activity following treatment, scores from both the HAT-QoL and IIEF questionnaires failed to reach statistical significance from baseline through post-treatment follow-up.^22^

Chaballout *et al* reported that 55% of participants experienced a greater-than-expected decline in sexual interest and 65% in sexual function in the long-term follow-up period.^23^ Although Franco-Rocha *et al* did not quantitatively assess sexual health, qualitative responses highlighted sexual function as a key concern, with one participant expressing the need for clinicians to recognise their anus as a sexual organ:

Yes, it was very important for me to have oncologists that realized that my anus serves as a sexual organ.^24^

#### Clinical guidelines and policy

Due to the rarity of anal cancer diagnosis, and the limited evidence base, anal cancer guidelines in the UK have historically been integrated within broader colorectal care guidelines.^30^

We identified four guidelines relevant to our review: two UK-based and two European guidelines. While our focus was primarily on UK guidance, European guidelines were included because they are currently referenced by UK professional bodies (ACPGBI and BHIVA) in the absence of more recent UK updates.

1. European AIDS Clinical Society (2025).^31^
2. European Society for Medical Oncology (ESMO) Clinical Practice Guidelines for Anal Cancer (2021).^32^
3. North of Scotland Clinical Management Guideline (2023).^33^
4. Royal College of Radiologists National Guidance for Volumetric Modulated Arc Therapy or Intensity-Modulated Radiation Therapy in Anal Cancer.^34^

Table 5 compares key aspects of HIV and anal cancer management across guidelines, including HIV-specific care, opportunistic infection prophylaxis, screening, cancer treatment (chemotherapy/radiotherapy), management of treatment-related toxicity, follow-up and long-term function and quality of life.

**Table 5:** Summary of guidance coverage across key guidelines for HIV and anal cancer care

	HIV-specific management	OI prophylaxis during cancer treatment	Anal cancer screening	Anal cancer-specific treatment management	Chemotherapy treatment	Radiotherapy treatment	Anal cancer toxicity	Anal cancer follow-up	Anal cancer long term function and QoL
EACS guidelines V12.0 October 2023	✔	✔	✔	X	X	X	X	X	X
ESMO Clinical Practice Guidelines for Anal cancer 2021	1	✔	✔	✔	✔	✔	✔	✔	1
North of Scotland Clinical Management Guideline 2021	X	X	X	✔	✔	1	✔	✔	*
RCR National Guidance for VMAT or IMRT in Anal Cancer (2024)	1	1	X	✔	X	✔	X	1	X

A tick (✔) indicates the domain is explicitly addressed in the guideline; (X) indicates it is not addressed; (1) refers to limited clinical detail. (*) Signifies the domain is implied but not clearly defined.

EACS, European AIDS Clinical Society; ESMO, European Society for Medical Oncology; IMRT, Intensity-Modulated Radiation Therapy; OI, opportunistic infection; QoL, Quality of Life; RCR, Royal College of Radiologists; VMAT, Volumetric Modulated Arc Therapy.

The included guidelines generally focus on either HIV or anal cancer, with minimal integration of both conditions. The most comprehensive treatment pathway, ESMO,^32^ acknowledges the importance of holistic care but provides limited practical guidance on managing short- and long-term effects in patients treated for anal cancer and does not include HIV-specific recommendations (table 5). While the guideline briefly references sexual and bowel function, it does not detail specific dysfunctional effects, psychosocial impacts or patient-reported experiences, nor offer practical guidance on preventing or managing these effects.

ESMO recommends follow-up with HIV medical teams and documentation of quality of life and late effects but notes that PRO measures are based on clinical experience and case reports rather than robust studies.^32^ No clear pathways for long-term care beyond tumour monitoring were identified for people living with HIV treated for anal cancer.

### Stakeholder consultation summary

Four stakeholders with lived experience of HIV and anal cancer treatment discussed the scoping review findings. While the identified studies acknowledged fatigue, pain and sexual well-being, stakeholders highlighted important gaps. Pain, they noted, was more complex than questionnaire data captured,^22 23^ encompassing both cancer-related discomfort and chemoradiotherapy treatment effects. When discussing preparedness for treatment, our stakeholders praised the information provided about the technical aspects of their chemoradiotherapy but, like Chaballout,^23^ found they felt unprepared for short- and long-term effects related to bowel health and sexual well-being.

Our stakeholders felt that important aspects of care specific to people living with HIV who require treatment for anal cancer, both in terms of their experience of illness and their healthcare needs and priorities, were currently underrepresented in research and absent in clinical guidelines. Consistent with Franco-Rocha’s findings on sexual well-being,^24^ stakeholders emphasised that healthcare providers often overlooked the anus as a sexual organ. While vaginal dilator use was routinely discussed to support intimacy after pelvic radiotherapy, conversations about anal sex were rarely addressed, leaving participants feeling uncertain and unsupported. This disconnect between clinician and patient priorities contributed to a sense that sexual well-being was dismissed, with attention narrowly focused on fertility.

Stakeholders called for more nuanced exploratory research, improved healthcare provider training, peer support networks and structured pretreatment and post-treatment guidance.

## Discussion

Dual stigma and siloed services between oncology and HIV care can create barriers for people living with HIV who are treated for anal cancer. Yet this population remains largely absent from research, with only three studies meeting inclusion criteria and none examining their experiences as a distinct group. These studies identify biopsychosocial and sexual challenges in the wider population treated for anal cancer that we argue may be compounded for people living with HIV. This review invites reflection on the structural influences that may contribute to this gap, examines the absence of survivorship guidance in clinical guidelines, and considers what the limited evidence suggests about physical, psychosocial and sexual health impacts. While the evidence is substantially lacking, this review is warranted to illuminate what remains invisible and encourage dialogue to improve support for this group.

### Structural and systemic factors that may contribute to limited research

The paucity of HIV-specific research in anal cancer likely reflects multiple structural and social factors. Anal cancer is relatively rare, which may limit research funding prioritisation.^35^ Additionally, both HIV and anal cancer are associated with social stigma. HIV stigma, rooted in historical associations with marginalised communities and misconceptions about transmission,^36 37^ can create environments where people both experience and fear judgement or discrimination. Anal cancer may also be stigmatised due to its association with sexual practices and human papillomavirus HPV infection.^38^ This dual stigma may create conditions that feel unsafe for both research participation and disclosure of HIV status to healthcare providers, contributing to underrepresentation in research, the development of interventions that do not reflect patients’ needs, and thus compromised quality of care.

### Physical health and functional impacts

The included studies reported short-term and long-term bowel side effects, symptoms of sexual dysfunction and pain affecting all anal cancer patients.^22^,^24^ These findings reflect broader trends in oncology, where patients have reported feeling unprepared for the physical and emotional impacts of both cancer treatment and survival, associated with limited discussions on late effects from healthcare professionals.^39^ Our stakeholder feedback highlighted that people living with HIV may face additional concerns, including potential effects of treatment on immune function and barriers to accessing multidisciplinary support for late effects. Stakeholders also noted that questionnaire-based measures may not fully capture the complexity of these experiences. However, the combined data make it difficult to determine whether HIV status modifies the severity or trajectory of these outcomes, as none of the studies examined HIV-specific differences. This gap reinforces the need for targeted research to understand how these challenges manifest and impact the quality of life for people living with HIV.

### Psychosocial health and stigma

Franco Rocha’s secondary analysis of LGBTQ+ survey data suggested that for people living with HIV, experiences of distress following anal cancer diagnosis varied.^24^ This is somewhat consistent with some of the HIV literature which has shown that some demonstrate resilience, potentially shaped by coping strategies developed during the early HIV epidemic when social exclusion, discrimination and mortality rates were high.^40^,^42^ For others, the historical collective trauma, associated with ongoing social disadvantage, chronic stress and mental and physical health challenges,^42^ may have heightened vulnerability to distress following an anal cancer diagnosis. Notably, the perception of both HIV and cancer as a ‘death sentence’ ^42 43^ may further amplify psychological distress when a person living with HIV is diagnosed with anal cancer. However, the included studies did not examine causal relationships or explicitly address dual stigma, nor did they analyse distress across intersecting social or demographic factors. It is therefore not possible to determine whether distress was attributable to HIV, anal cancer or other contextual influences. Understanding what promotes resilience and what contributes to vulnerability remains essential for developing equitable, responsive psychosocial interventions for people living with HIV.

### Sexual well-being

Greater-than-anticipated impacts on sexual desire and function^22^ may reflect inadequate pretreatment counselling about potential late effects.^39 44^ Anal cancer is futher shaped by broader societal taboos that can inhibit open discussion of the anus, anal sex and sexual health.^30^ For people living with HIV, this taboo could be compounded by HIV-related stigma. Broader literature shows that HIV care has often prioritised risk reduction over intimacy and pleasure.^45^ Indeed, historical messaging around HIV has frequently been shaped by fear, centring on transmission and mortality, rather than supporting sexual well-being.^45 46^ These combined factors may create barriers to clinical discussion about sexual well-being.^44^ Our stakeholders emphasised that healthcare providers must acknowledge diverse sexual practices, including anal sex, and recognise that sexual practices are not dependent on sexual identity or gender. Targeted research is needed to understand how anal cancer treatment affects sexual well-being for people living with HIV and to develop interventions supporting physical recovery and pleasurable sexual experiences.

### Limitations

Only three studies met the inclusion criteria, all of which aggregated data from people living with HIV and those without HIV. Additionally, research articles may have been missed where potential HIV status was not included, making the invisibility of this population even more profound. While formal critical appraisal was beyond the scope of this scoping review, methodological concerns were noted, including inconsistent statistical reporting. Relevant evidence may also exist in grey literature or non-English publications. The studies relied on quantitative tools that stakeholders felt did not capture the nuances of lived experiences. The review is therefore limited by the inability to identify HIV-specific patterns. However, despite these gaps, the review identifies areas of potential importance (bowel symptoms, anticipated effects of treatment, pain and sexual well-being) for all people with anal cancer, but highlights the need for further exploration, specifically for people living with HIV.

### Implications for research and clinical care

Despite the limited number of studies, this review identifies several key messages:

1. People living with HIV and treated for anal cancer are underrepresented in research, making their experiences largely invisible.
2. Existing clinical guidelines focus on biomedical outcomes and offer little guidance for survivorship, psychosocial support or sexual well-being.
3. Structural and social factors, including stigma, fragmented care and research priorities, contribute to gaps in both evidence and care.
4. There is a pressing need for targeted research that captures the lived experiences of people living with HIV; these insights could then inform inclusive clinical guidance in the support of patient-centred care.

## Conclusions

This review has identified a significant gap: people living with HIV who are treated for anal cancer remain largely invisible in the research literature. While the scarcity of evidence limits definitive conclusions, it raises important questions about how living with both conditions may differentially affect quality of life, how survivorship is prioritised within clinical guidelines, and how structural factors, including stigma and fragmented care pathways, shape both research and practice. Research that centres the lived experiences of people living with HIV is essential to understand whether and how treatment impacts differ for this population, and to inform integrated, equitable care models that address their complex needs.

## Supplementary material

10.1136/bmjopen-2025-114180 online supplemental file 1

10.1136/bmjopen-2025-114180 online supplemental file 2

10.1136/bmjopen-2025-114180 online supplemental file 3

10.1136/bmjopen-2025-114180 online supplemental file 4

## Data Availability

Data are available on reasonable request. All data relevant to the study are included in the article or uploaded as supplementary information.
