# Supplementary material for: Lived experiences of cancer care for people living with HIV who are treated for anal cancer: a scoping review
Source: BMJ Open. 2026 Mar 30;16(3):e114180. doi: 10.1136/bmjopen-2025-114180 (PMC13052807; doi:10.1136/bmjopen-2025-114180)
Supplement: online supplemental file 2 [file bmjopen-16-3-s002.pdf]

Interface – EBSCOhost Research Databases; Advanced Search; Databases – CINAHL Ultimate; MEDLINE Complete; Psychology and Behavioral Sciences Collection; GreenFILE; MEDLINE; eBook Collection (EBSCOhost); CINAHL; Kortext eBook Catalogue London, Kent, Surrey & Sussex; London Health Libraries

Query	Limiters/ Expanders	Results
**S9 – patient reported outcomes**	Exp- RW; SM-Prox	170,042
**S8 – psychosocial wellbeing OR psychosocial OR emotional wellbeing OR emotional OR psychological**	Exp- RW; SM-Prox	4,441,259
**S7 – satisfaction OR experience OR perception OR view**	Exp- RW; SM-Prox	6,853,943
**S6 – life quality**	Exp- RW; SM-Prox	36,055
**S5 – health related quality of life OR HRQoL OR quality of life OR QoL**	Exp- RW; SM-Prox	1,915,638
**S4 – patient centred care**	Exp- RW; SM-Prox	103,364
**S3 – survivorship**	Exp- RW; SM-Prox	77,499
**S2 – quality of life**	Exp- RW; SM-Prox	1,909,327
**S1 – lived experience**	Exp- RW; SM-Prox	73,787

Query	Limiters/ Expanders	Results
**S24 – S1 AND S15 AND S21**	SM-Prox	0
**S23 – S2 AND S15 AND S21**	SM-Prox	133
**S22 – S10 AND S15 AND S21**	SM-Prox	45
**S21 – S16 OR S17 OR S18 OR S19 OR S20**	SM-Prox	11,402
**S20 – Anal neoplasms**	SM-Prox	187
**S19 – Anal tumors**	SM-Prox	380
**S18 – Anal malignancy**	SM-Prox	112
**S17 – anal cancer OR anal squamous cell carcinoma**	SM-Prox	11,007
**S16 – anal cancer**	SM-Prox	9,933
**S15 – S11 OR S12 OR S13 OR S14**	SM-Prox	1,207,617
**S14 – HIV**	SM-Prox	1,168,720
**S13 – people living with HIV**	SM-Prox	52,621
**S12 – HIV positive**	SM-Prox	142,799
**S11 – human immunodeficiency virus**	SM-Prox	534,418
**S10 – S2 AND S5 AND S8**	SM-Prox	415,663

Query	Limiters/ Expanders	Results
**S37 – S15 AND S17 AND S36**	SM-Prox	161
**S36 – qualitative research OR qualitative study OR qualitative methods OR interview**	SM-Prox	2,135,240
**S35 – S3 AND S15 AND S16**	SM-Prox	8
**S34 – S15 AND S21 AND S33**	SM-Prox	4
**S33 – "Late effects"**	SM-Prox	20,199
**S32 – S15 AND S21 AND S31**	SM-Prox	133
**S31 – "Quality of life"**	SM-Prox	1,678,514
**S30 – S9 AND S15 AND S21**	SM-Prox	20
**S29 – S15 AND S21 AND S28**	SM-Prox	4
**S28 – S4 OR S27**	SM-Prox	113,989
**S27 – person centred care**	SM-Prox	16,670
**S26 – S8 AND S15 AND S21**	SM-Prox	159
**S25 – S3 AND S10 AND S15 AND S21**	SM-Prox	0

Key

Exp – RW = Expanders – Apply related words

SM – Prox = Search modes – Proximity
