# Supplementary material for: Lived experiences of cancer care for people living with HIV who are treated for anal cancer: a scoping review
Source: BMJ Open. 2026 Mar 30;16(3):e114180. doi: 10.1136/bmjopen-2025-114180 (PMC13052807; doi:10.1136/bmjopen-2025-114180)
Supplement: online supplemental file 3 [file bmjopen-16-3-s003.pdf]

The ROYAL MARSDEN
NHS Foundation Trust

Making Sense of the Data: Connecting Lived Experience to Research

Emma Hainsworth & Christine Addington

NHS

Overview

Aims:

Provide an overview of the findings from our literature searches

Explore how this fits in with your experience

Identify gaps and next steps

Structure:

5 mins intro with icebreaker

Presentation of the themes

Around 20 minutes of discussion on different areas found in the research

Why we need your help

Filling the gaps. Research and clinical guidelines often focus on treatment outcomes but lack insight into lived experiences

Your involvement helps:

- Identify missing perspectives
- Ensure findings are meaningful and applicable
- Shape recommendations that will inform real change
- Strengthen the connection between research, policy, and lived experience

Starting the conversation

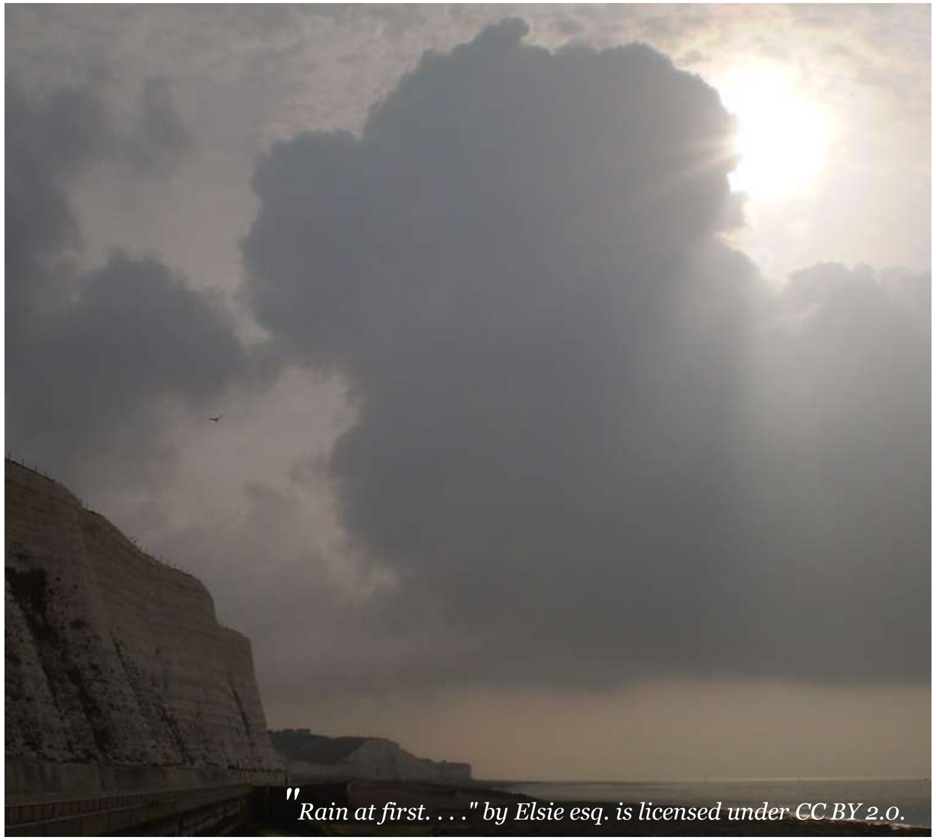

"Rain at first. . . ." by Elsie esq. is licensed under CC BY 2.0.

- If you were to describe your experience living with HIV and undergoing treatment for anal cancer in terms of weather, what imagery or sensations would you use?
- How do these weather metaphors capture the complexity of your journey?
- Examples: Has it felt stormy, filled with uncertainty? Like a foggy morning with low visibility but a hint of hope?

Key Themes in Research

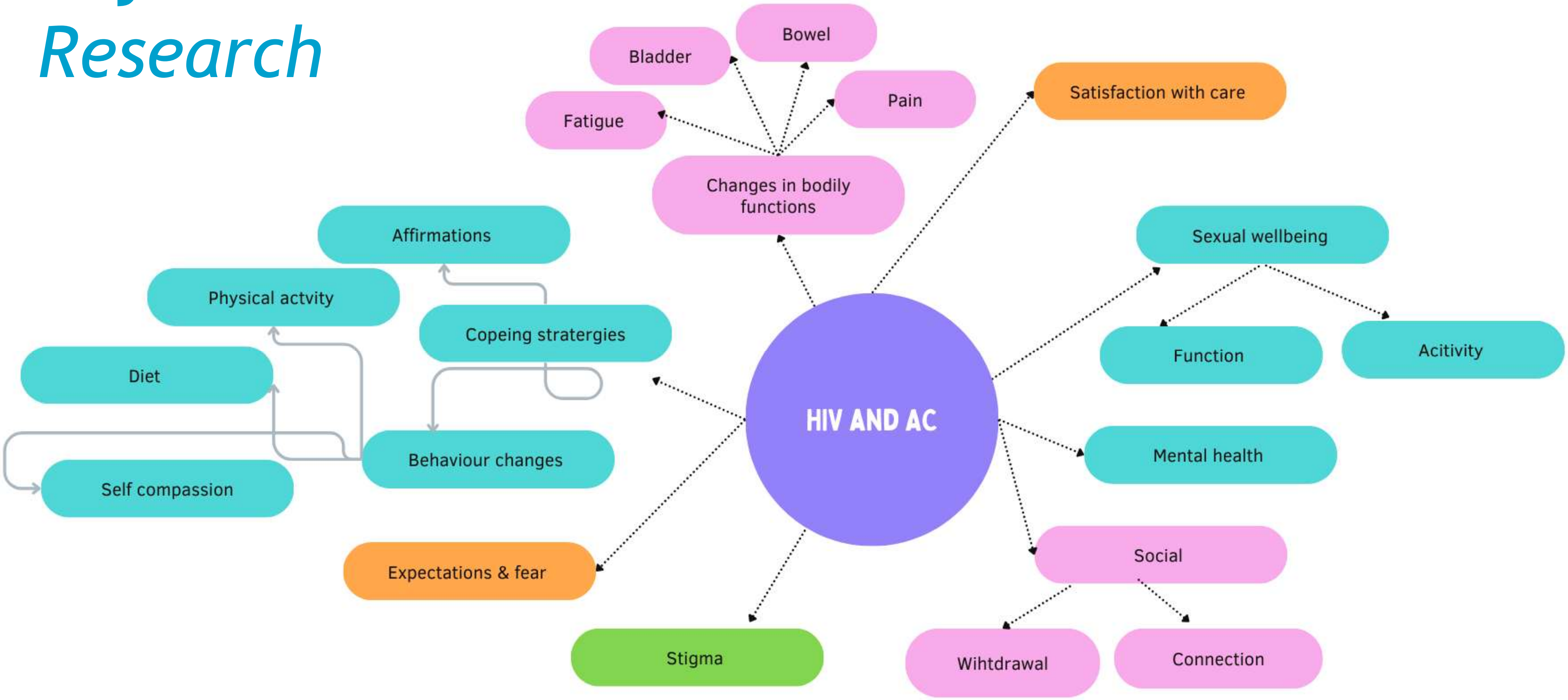

Changes in bodily function, social & mental well-being

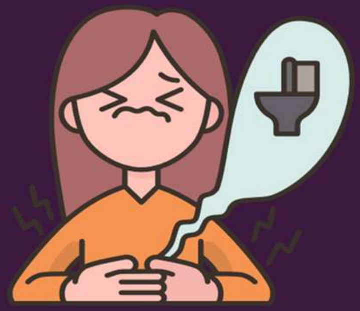

Short-term side effects from treatment (such as **pain**, **bowel issues**, and **fatigue**) were reported as **worse** than expected.

Long-term symptoms (e.g., neuropathy, diarrhoea, memory issues) **persisted**, affecting **mental health** and **social well-being**.

Does this reflect your experience ?

Sexual well-being & relationships

Treatment had a significant impact on intimacy, with many experiencing changes in sexual function and self-esteem. Some adapted their relationships, while others **withdrew** from intimacy due to pain, fear, or feelings of undesirability.

People from the LGBTQ+ community were more likely to report relationship breakdowns.

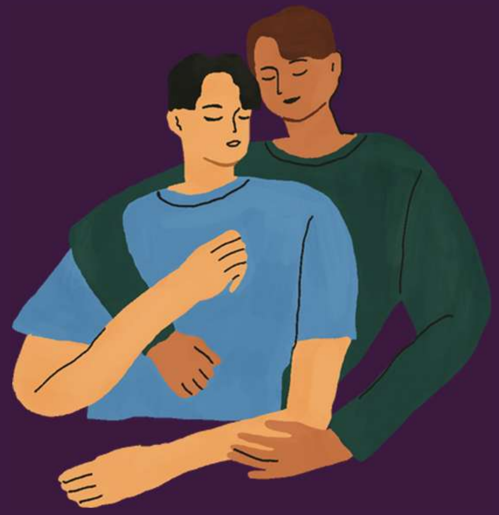

Stigma & disclosure

Many chose not to disclose their diagnosis or treatment side effects to friends, family, or colleagues due to fear of stigma.

Those living with HIV often faced additional challenges in disclosure.

Some people reported that healthcare interactions **reinforced** feelings of **shame.**

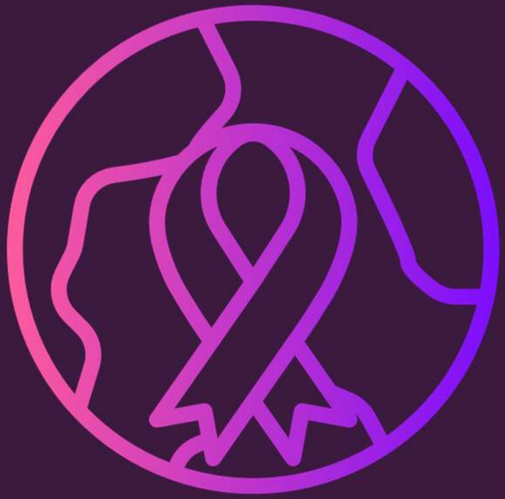

Satisfaction with care & cultural competence

Around half of participants felt their healthcare providers were culturally competent.

However, people from LGBTQ+ community reported feeling misunderstood or overlooked in their care.

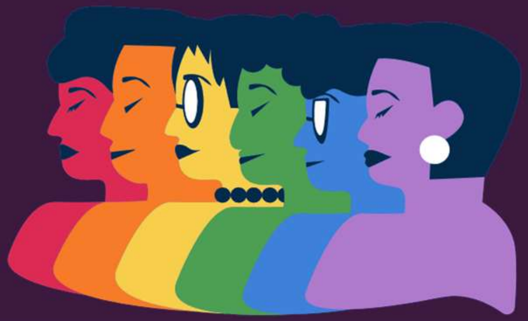

Strategies for managing

Many described seeing life differently post-treatment, with some adopting **healthier habits.**

Social support played a crucial role, those with **fewer close connections** reported **higher** levels of **distress.**

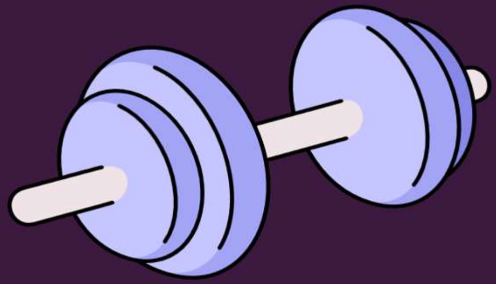

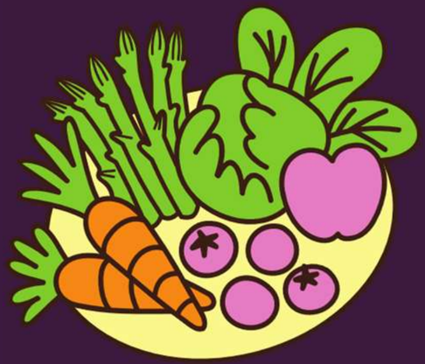

Future Research & Policy Recommendations –

The papers suggest a need for **peer support programs**, better integration of psychosocial care in guidelines, and further research into the **neuropsychological** effects of treatment.

Training for healthcare professionals was also highlighted as a key area for improvement.

Is there an aspect of your experience of anal cancer missing?

- What resonated most with you?
- Are there any missing perspectives or themes we should explore further?
- What findings feel most important for healthcare services and policies?
- Is there anything that needs clearer communication or further research?"

- Changes in bodily function, social & mental well-being
- Sexual well-being & relationships
- Stigma & disclosure
- Satisfaction with care & cultural competence

Next Steps:

- Refining findings based on participant insights.
- Exploring strategies for knowledge translation.
- Identifying priorities for future research and policy improvements.

Feedback & Thank You!

Linking Lived Experience of Anal Cancer Treatment to Research
