# Supplementary material for: Lived experiences of cancer care for people living with HIV who are treated for anal cancer: a scoping review
Source: BMJ Open. 2026 Mar 30;16(3):e114180. doi: 10.1136/bmjopen-2025-114180 (PMC13052807; doi:10.1136/bmjopen-2025-114180)
Supplement: online supplemental file 4 [file bmjopen-16-3-s004.pdf]

Linking Lived Experience of Anal Cancer Treatment to Research

Thank you for taking the time to complete this survey! Your feedback helps us assess how effectively we shared up-to-date research and identify ways to improve. All responses are completely anonymous, so please share your honest

1. How relevant did you find the workshop discussions to your experience of being treated for anal cancer?

- Extremely relevant ○
- Somewhat relevant ○
- Neutral ○
- Not very relevant ○
- Not relevant at all ○

2. How clear were the key findings presented?

- Very clear ○
- Somewhat clear ○
- Somewhat unclear ○
- Very unclear ○

3. Did you feel you had the chance to contribute to the discussions ?

- Yes, definately ○
- Somewhat ○
- Not at all ○

4. Do you feel the project is going in the best direction that relates to your lived experience?

5. What was the most valuable part of the workshop for you?

6. How comfortable did you feel sharing your thoughts during the session?

Very comfortable	Somewhat comfortable	Neither comfortable nor uncomfortable	Somewhat uncomfortable	Very uncomfortable
○	○	○	○	○

7. Do you feel that your insights were listened to and considered?

Strongly agree	Agree	Neither agree nor disagree	Disagree	Strongly disagree
○	○	○	○	○

8. How useful do you think this workshop will be in translating findings into healthcare services

Extremely useful	Somewhat useful	Very useful	Extremely not useful	Somewhat not useful
○	○	○	○	○

9. Based on your responses or other areas is there anything you would like to add

This content is neither created nor endorsed by Microsoft. The data you submit will be sent to the form owner.

Microsoft Forms
