# Supplementary material for: Lived experiences of cancer care for people living with HIV who are treated for anal cancer: a scoping review
Source: BMJ Open. 2026 Mar 30;16(3):e114180. doi: 10.1136/bmjopen-2025-114180 (PMC13052807; doi:10.1136/bmjopen-2025-114180)
Supplement: Supplementary file 5 [file bmjopen-2025-114180.reviewer_comments.pdf]

PEER REVIEW HISTORY

BMJ Open publishes all reviews undertaken for accepted manuscripts. Reviewers are asked to complete a checklist review form and are provided with free text boxes to elaborate on their assessment. These free text comments are reproduced below.

ARTICLE DETAILS

Title (Provisional)

The lived experiences of cancer care for people living with HIV who are treated for anal cancer: a scoping review

Authors

Addington, Christine; Davies, Nigel; Howell, Paul; Cruickshank, Susanne; Hainsworth, Emma

---

VERSION 1 - REVIEW

**Reviewer**	**1**
**Name**	**Cao, Gaoyang**
**Affiliation**	**Zhejiang University**
**Date**	**04-Dec-2025**
**COI**	**None**

Recommendation: Minor Revision

This scoping review addresses a clinically important yet substantially understudied topic—the lived experiences of people living with HIV (PLWH) who undergo treatment for anal cancer. Given the disproportionate burden of anal cancer among PLWH and the significant functional, psychosocial, and sexual health impacts of chemoradiation, the manuscript fills an important research gap. The methodology is generally rigorous, incorporating PRISMA-ScR guidelines, a clearly justified PCC framework, and a valuable stakeholder consultation component that strengthens the relevance and interpretability of the findings.

The manuscript is comprehensive and well structured. However, a few revisions would enhance clarity, transparency, and the overall coherence of the review.

1. Clarify and strengthen explanation regarding the limited number of included studies

Only three studies were included in the final review. While this reflects the scarcity of evidence rather than methodological shortcomings, the manuscript would benefit from a more explicit discussion of:

Why the evidence base is so limited

How this paucity of research reinforces the necessity of the review

Potential structural reasons (e.g., stigma, underdiagnosis, lack of qualitative research)

Enhancing this will help readers understand the importance and validity of the review despite the low number of included articles.

2. Provide more detail on study selection, particularly exclusion decisions

The manuscript could be strengthened by clarifying:

How “lived experience” was operationalized.

Specific examples of common types of studies excluded and why.

This will improve reproducibility and transparency.

3. Pre-treatment details should be further illustrated

The manuscript notes that one of the included studies involved benign adenoma not amenable to endoscopic removal. To improve clarity, including representative colonoscopic images or describing the endoscopic findings more explicitly would help demonstrate why endoscopic resection was not feasible.

4. Improve clarity of guideline analysis

The guideline mapping section is valuable but would benefit from:

Clearer description of the criteria used to evaluate guideline content

A short table summarizing guideline gaps across domains (sexual health, psychosocial care, survivorship, etc.), which will improve readability.

5. Minor editorial revisions

Ensure consistency in terminology (e.g., “people living with HIV” vs “HIV-positive individuals”).

Abbreviations should be defined at first use.

A small number of sentences could be tightened for clarity; a light language polish would be beneficial.

Conclusion

This manuscript offers a valuable and much-needed synthesis of the very limited evidence surrounding the lived experience of PLWH undergoing treatment for anal cancer. The study is thoughtfully designed and well executed. With minor revisions to improve clarity, transparency, and presentation, it will make a meaningful contribution to the literature and to clinical practice.

**Reviewer**	**2**
**Name**	**Sodergren, Sam**

**Affiliation**	**University of Southampton**
**Date**	**11-Dec-2025**
**COI**	**None**

Thank you for the opportunity to review this manuscript on such an under-researched area which certainly deserves more attention.

Please see below recommendations for improvement:

A particular strength of this work is the involvement of people with lived experience to review the work and interpretations.

Abstract

Purpose

The was in which the final sentence of this section is presented is indicative of results rather than study aim so consider reframing as follows: "This review aims to highlight the …..

Methods

Consider restructuring this section to add clarity. I was unsure at first what the focus of the searches actually was. You could firstly identify the search strategies and reserve information about the number of studies captured (n=3) (and information about number of guideline documents extracted – this is not clear in the results) for the results section. I would recommend clearly outlining the inclusion criteria for studies – those looking at anal cancer and HIV with a focus on lived experiences and guidelines for this patient group?

Introduction

You refer to the health outcomes of patients with anal cancer with HIV, a discussion of health-related quality of life (HRQoL) outcomes could be outlined. Although biomedical outcomes are the primary focus of clinical trials, inclusion of HRQOL measures are recommended to support labelling claims. However, as noted, patient-reported outcomes are not commonly reported.

When citing survival statistics, it would be advisable to refer to older patients (i.e., above the 15-44 years for whom anal cancer is particularly rare).

Methods

Good overview (and careful consideration) of the research question identification and search strategies in this section provided.

In terms of population, I note that you include people living with HIV and diagnosed with anal cancer. Did this also include samples of patients with anal cancer of whom some have HIV (i.e., mixed samples?) although I appreciate data collection for HIV status might be an issue and thus be under-reported (i.e., not all studies collect information on this)? Note this is something that could have been discussed in the discussion.

In table 2, the inclusion for research studies is specified as published within the last year yet in the text, no time limit was specified.

Including international guidelines (i.e., not just those applied to the UK) might have been still informative. I note that the EU guidelines were included.

PPIE input: Did patients with lived experience receive any training in how to help you evaluate the review results?

I could not locate a table outlining the search terms (I am wondering whether this has been cut off in the proof I have to review?). I see that this is presented as an appendix but it is still not overly clear. I would recommend a full outline of the search terms as it is not clear whether the terms HIV and anal cancer (and their synonyms) were required for the title, abstract and therefore whether the searches were exclusively on HIV and anal cancer? I am just wondering whether including anal cancer in general (without HIV in the title and abstract) would have resulted in more hits and then through reading the demographics and results you could ascertain whether the sample included those with HIV – indeed, looking at the results it appears that the samples of three studies included are not exclusively patients with anal cancer and with HIV.

Results

p.10 include the year for the European Organisation for Research and Treatment of Cancer Quality of Life Group's definition of HRQOL (2018?). Check also whether this is the best citation to include.

The results presented are for the sample as a whole rather than a sub-group of patients with anal cancer and HIV.

Include references alongside measures (e.g., EORTC QLQ-C30, QLQ-ANL27, QLQ-CR29, DISC).

The results section could benefit from being considerably more concise, especially with respect to the outcome measure section.

Involvement of patients with lived experience strengthened the results section.

Unfortunately, due to the scarcity of research and the presentation of results for patients with anal cancer without HIV, it is not clear the extent to which the results are specific to the patient group of interest.

Discussion

The discussion reads rather like an extension to the results section and would benefit from being focused on the key messages that have come this work and this is not overly clear currently.

VERSION 1 - AUTHOR RESPONSE

Source	Comment No.	Manuscript Section	Comment / Issue Raised	Author Response
**Reviewer 1**	1	Discussion	Clarify and strengthen explanation regarding the limited number of included studies Only three studies were included in the final review. While this reflects the scarcity of evidence rather than methodological shortcomings, the manuscript would benefit from a more explicit discussion of:<br>Why the evidence base is so limited<br>How this paucity of research reinforces the necessity of the review<br>Potential structural reasons (e.g., stigma, underdiagnosis, lack of qualitative research)<br>Enhancing this will help readers understand the importance and validity of the review despite the low number of included articles.	Thank you for this comment. We are certain that addressing it has strengthened our manuscript. We have expanded the discussion to highlight why the evidence is so sparse and how this reinforces the necessity of our review. We also note several potential structural factors, including stigma, underdiagnosis, and the historical lack of qualitative research in this area.<br><br>By including these points, we hope to emphasize the importance of this underrepresented population and the need to address the unmet needs of this underserved community.
**Reviewer 1**	2	Methods	Provide more detail on study selection, particularly exclusion decisions<br>The manuscript could be strengthened by clarifying:<br>How “lived experience” was operationalized.<br>Specific examples of common types of studies excluded and why.<br>This will improve reproducibility and transparency.	Thank you for this comment. We have included text explaining how lived experience was operationalised, added in the Methods section under Research question identification.
**Reviewer 1**	3	?	Pre-treatment details should be further illustrated<br><br>The manuscript notes that	We believe this comment may have been included in error.

Source	Comment No.	Manuscript Section	Comment / Issue Raised	Author Response
			one of the included studies involved benign adenoma not amenable to endoscopic removal. To improve clarity, including representative colonoscopic images or describing the endoscopic findings more explicitly would help demonstrate why endoscopic resection was not feasible.	
Reviewer 1	4	Findings (clinical guidelines)	Improve clarity of guideline analysis<br><br>The guideline mapping section is valuable but would benefit from:<br>Clearer description of the criteria used to evaluate guideline content<br>A short table summarizing guideline gaps across domains (sexual health, psychosocial care, survivorship, etc.), which will improve readability.	Thank you for this comment. Our intent was not to formally evaluate the guidelines, but rather to provide an overall descriptive overview of their content, which we have clarified in the manuscript.<br><br>As shown in the final column of Table 3, we indicate which guidelines reference domains of health-related quality of life. As can be seen, only the ESMO and CMG guidelines mention QoL domains, and the text specifies the particular areas they cover.<br><br>Under the Research section – Clinical guidelines and policy, a summary of the QoL domains referenced and what these entail.<br><br>Overall, we hope to demonstrate an absence of detailed QoL domains across most clinical guidelines, and where QoL is mentioned, it remains non-specific and limited in scope.

Source	Comment No.	Manuscript Section	Comment / Issue Raised	Author Response
**Reviewer 1**	5	Throughout	Minor editorial revisions<br><br>Ensure consistency in terminology (e.g., “people living with HIV” vs “HIV-positive individuals”).<br>Abbreviations should be defined at first use.<br>A small number of sentences could be tightened for clarity; a light language polish would be beneficial	We thank the reviewer for this comment. We have revised the manuscript to ensure consistent terminology throughout (using “people living with HIV”) and have defined all abbreviations at first use. Regarding language polishing, we have edited the abstract, which we felt was the main section requiring clarification, and believe the remainder of the manuscript is clear and appropriate.

Source	Comment No.	Manuscript Section	Comment / Issue Raised	Author Response
**Reviewer 2**	1	Abstract	The was in which the final sentence of this section is presented is indicative of results rather than study aim so consider reframing as follows: "This review aims to highlight the …..	Thank you for your feedback. We have now corrected the text to read as follows: "This scoping review aims to identify existing evidence…."
**Reviewer 2**	2	Abstract (Methods)	Methods<br>Consider restructuring this section to add clarity. I was unsure at first what the focus of the searches actually was. You could firstly identify the search strategies and reserve information about the number of studies captured (n=3) (and information about number of guideline documents extracted – this is not clear in the results) for the results section. I would recommend clearly outlining the inclusion criteria for studies – those looking at anal cancer and HIV with a focus on lived experiences and guidelines for this patient group?	The abstract has been re-structured in line with BMJ Open requirements and under new headings which we believe now provides the clarity requested
**Reviewer 2**	3	Intro/Background	1) You refer to the health outcomes of patients with anal cancer with HIV, a discussion of health-related quality of life (HRQoL) outcomes could be outlined. Although biomedical outcomes are the primary focus of clinical trials,	1. Thank you, we have edited the introduction to include this important point<br><br>2. We have now included age group over 50; "While the prognosis for anal cancer is generally favourable, with around 70% of people aged 15–44 surviving beyond 10 years and 55–65% of people over 50 surviving five years"

Source	Comment No.	Manuscript Section	Comment / Issue Raised	Author Response
			inclusion of HRQOL measures are recommended to support labelling claims. However, as noted, patient-reported outcomes are not commonly reported.<br><br>2. When citing survival statistics, it would be advisable to refer to older patients (i.e., above the 15-44 years for whom anal cancer is particularly rare).	
Reviewer 2	4	Methods	In terms of population, I note that you include people living with HIV and diagnosed with anal cancer. Did this also include samples of patients with anal cancer of whom some have HIV (i.e., mixed samples?) although I appreciate data collection for HIV status might be an issue and thus be under-reported (i.e., not all studies collect information on this)? Note this is something that could have been discussed in the discussion.	Please see clarification provided to comment 8 on the Search Strategy below
Reviewer 2	5	Methods Table 2	The inclusion for research studies is specified as published within the last year yet in the text no time limit was specified	Thank you for spotting this. We have now edited this oversight so that timeframes are consistent in text and in table.
Reviewer 2	6	Methods	Including international guidelines (i.e., not just those applied to the UK) might have been still	We appreciate the reviewer's comment. Our project is situated in the UK context, where resourcing

Source	Comment No.	Manuscript Section	Comment / Issue Raised	Author Response
			informative. I note that the EU guidelines were included.	defines how person-centred care can be delivered. EU guidelines were included because of the lack of updated UK-specific guidance, providing the most relevant international context for informing future work, while broader international guidelines were considered less directly applicable.
**Reviewer 2**	7	Methods	PPIE input: Did patients with lived experience receive any training in how to help you evaluate the review results?	Patients with lived experience did not receive formal training in analysis; however, they were provided with a briefing document outlining the review objectives and methods, and all had prior experience contributing to service evaluation work, enabling them to provide informed input on the findings.
**Reviewer 2**	8	Methods	I could not locate a table outlining the search terms (I am wondering whether this has been cut off in the proof I have to review?). I see that this is presented as an appendix but it is still not overly clear.<br><br>1) I would recommend a full outline of the search terms as it is not clear whether the terms HIV and anal cancer (and their synonyms) were required for the title, abstract	We thank the reviewer for this comment and hope we have clarified this.<br><br>1) The table presented in text is intended as an illustrative example of a single concept domain (in this case, quality of life and its related synonyms), rather than a full representation of the entire search strategy.

Source	Comment No.	Manuscript Section	Comment / Issue Raised	Author Response
			and therefore whether the searches were exclusively on HIV and anal cancer?<br><br>2) I am just wondering whether including anal cancer in general (without HIV in the title and abstract) would have resulted in more hits and then through reading the demographics and results you could ascertain whether the sample included those with HIV – indeed, looking at the results it appears that the samples of three studies included are not exclusively patients with anal cancer and with HIV.	The appendix shows our full list across all PCC terms. We have reorder this to help with ease of reading<br><br>2) We have verified that the search strategy did not restrict title and abstract fields to require both HIV and anal cancer terms.
Reviewer 2	9	Results	p.10 include the year for the European Organisation for Research and Treatment of Cancer Quality of Life Group's definition of HRQOL (2018?). Check also whether this is the best citation to include.	Thank you. In keeping with your suggestion of making the results more concise this has now been removed as it was felt that it did not give anyfuther as we define in the methods.
Reviewer 2	10	Results	The results presented are for the sample as a whole rather than a sub-group of patients with anal cancer and HIV.	
Reviewer 2	11	Results	Include references alongside measures (e.g.,	Thank you for spotting this oversight. These have now

Source	Comment No.	Manuscript Section	Comment / Issue Raised	Author Response
			EORTC QLQ-C30, QLQ-ANL27, QLQ-CR29, DISC).	been added. Unfortunately, no date was provided for the EORTC questionnaires, so this has been indicated as [NR] (not reported). If this is not in line with journal convention, we would be happy to amend this accordingly.
Reviewer 2	12	Results	The results section could benefit from being considerably more concise, especially with respect to the outcome measure section.	Thank you for your guidance. The Results section has been substantially revised to improve clarity and conciseness, with particular attention to the outcome measures section. Redundant descriptions have been removed, overlapping content streamlined, and results synthesised thematically to reduce repetition while preserving key findings and methodological transparency.
Reviewer 2	13	Results	Unfortunately, due to the scarcity of research and the presentation of results for patients with anal cancer without HIV, it is not clear the extent to which the results are specific to the patient group of interest.	We agree with this observation. The scarcity of research specifically focused on people living with HIV with anal cancer, and the frequent aggregation of results for anal cancer populations without stratification by HIV status, limits the extent to which findings can be attributed solely to the population of interest. This limitation has now been explicitly acknowledged in the Results and Discussion sections, and we have

Source	Comment No.	Manuscript Section	Comment / Issue Raised	Author Response
				highlighted the need for future research to report outcomes stratified by HIV status to better inform patient-centred care for this group.
Reviewer 2	14	Discussion	The discussion reads rather like an extension to the results section and would benefit from being focused on the key messages that have come this work and this is not overly clear currently.	We thank the reviewer for this comment. The Discussion has been revised to reduce repetition of the Results, add a “Key Points” section, and focus on interpretation and synthesis, highlighting gaps in evidence, dual stigma, and implications for patient-centred care. Despite the limited HIV-specific data, the review remains important to illuminate potential impacts and highlight the need for dedicated research on people living with HIV treated for anal cancer.
Note from authors		Reference list		The reference list has been updated to correct formatting and to include additional citations in accordance with the reviewer’s feedback.
